# Mental Health of Muslim Nursing Students in Thailand

**DOI:** 10.5402/2012/463471

**Published:** 2012-06-25

**Authors:** Paul Ratanasiripong

**Affiliations:** Department of Advanced Studies in Education and Counseling, California State University, Long Beach, 1250 Bellflower Boulevard, Long Beach, CA 90840, USA

## Abstract

The purpose of this research was to explore the mental health and well-being of Muslim nursing students in Thailand. Specifically, the study investigated the factors that impact anxiety and depression among Muslim nursing students. This cross-sectional research was conducted with a half sampling method of Muslim undergraduate students who were studying at a public nursing college in Thailand. From the 220 self-identified Muslim nursing students, 110 were sampled for this study, representing 14% of the total nursing students at this college. Results indicated a moderate prevalence of anxiety and high prevalence of depression among Muslim nursing students. Stress (*β* = .42) was positively associated with anxiety, while self-esteem (*β* = -.42) was negatively associated with anxiety; together this model accounted for 46% of the variance in anxiety. Self-esteem (*β* = -.41) and social support (*β* = -.17) were negatively associated with depression, while stress (*β* = .37) was positively correlated with depression; together this model accounted for 57% of the variance in depression. Recommendations were given to help train Muslim nursing students to be competent nurses with good mental health and well-being who will succeed and contribute to the nursing profession.

## 1. Introduction

Over the past decade, numerous studies have demonstrated the need for a more culturally and religiously sensitive patient care from nurses and nursing students [1–5]. Additional studies have offered recommendations on how to provide care tailored to the needs of specific religious and cultural groups [6–11].

Currently, Muslims are one of the fastest growing religious groups in the world with over 1.5 billion people [12]. Increasingly, patients seen in hospitals, at outpatient clinics, and during home visits are Muslim. In response to this population increase, studies have been conducted to further our understanding of the specific needs of the Muslim patients [13–16]. Several recent books have also addressed how to specifically care for Muslim patients [17, 18].

Although it is important to train nurses and nursing students to be culturally and religiously sensitive, it is even more important for nursing professionals to understand both the religious and cultural standards of Islam before treating Muslim patients. Muslim patients appreciate not having to explain that they have to pray five times per day, eat only certain foods (halal), not eat certain foods (haram), or fast during the month of Ramadan. Muslim patients also prefer to be cared for by nurses and medical professionals of the same gender. In addition to familiarizing**  **nursing students with the religious and cultural conventions of Muslim patients, it is important, as per Narayanasamy and Andrews' recommendations [19], to recruit and train Muslim nursing students to better meet the needs of the growing Muslim patient population globally.

Beyond recruiting and training, it is imperative that nurse educators facilitate the retention and professional development of Muslim nursing students. As the shortage of nurses is an increasing problem in many countries around the world, early intervention and retention efforts by nurse educators could help train Muslim nursing students not only to be competently skilled nurses, but also to be cognizant of their own mental well-being so that they will enjoy their profession for a lifetime.

### 1.1. Anxiety and Depression

An increase in the frequency and severity of psychological symptoms among college students in the past decade has been well-documented [20–23]. Increasingly, college students in various countries are reporting experiences of anxiety and depression [24–27]. Studies have shown that mental health issues negatively impact the academic performance and retention of college students [28–30]. In fact, the American College Health Association reported that depression and anxiety are two of the top ten impediments to college students' academic performance [28]. Backels and Wheeler also found that most faculty feel that mental health issues negatively impact the college students' academic performance [29]. In a recent study in Southeast Asia, O'Brien et al. reported that the depression and stress among college students negatively impacted their academic performance and social involvement [30].

Nursing students in particular face many challenges in the profession including stress and fatigue from nursing school, emotional exhaustion, nursing shortage, and increasing employment turnover rates [31–34]. Their struggles with anxiety and depression have been documented in several studies. Several researchers reported a high prevalence of anxiety among nursing students in the United States and Iran [35–37]. Other studies found a high prevalence of depression among nursing students in Iran and Thailand [38, 39].

In order to understand anxiety, one of the major hindrances to students' mental health and well-being, studies have examined its various influences. Studies have demonstrated that the stress level college students experience contribute to the anxiety symptoms [40]. Bunevicius et al. also indicated that the severity of symptoms of anxiety is positively related to the stress vulnerability among medical students [41]. On the other hand, Suliman and Halabi found that self-esteem was negatively correlated with anxiety among nursing students [42]. In addition, studies have also discovered the negative association between social support and anxiety among nurses [43]. 

Depression, the second hindrance to mental health and well-being among nursing students, has been shown to increase as stress levels increase. Ross et al. reported in their study that stress accounted for 10.5% of the variance in depression among nursing students [39]. Other studies reinforced the connection between increasing stress and increasing depression among medical residents in Argentina [44] and among employees in China [45]. On the other hand, factors that have contributed to the reduction in depressive symptoms include higher levels of support and higher levels of self-esteem [39, 46]. Nirattharadorn, Phancharoenworakul, Gennaro, Vorapongsathorn, and Sitthimongkol found that social support and self-esteem had significant influence in reducing depression [47]. Similarly, Shikai et al. found self-efficacy to be negatively associated with depression [48].

### 1.2. Muslim Nursing Students

Several studies have been performed to understand the mental health of nursing students [48–51]. However, no research has been conducted specifically on the mental health of Muslim nursing students. With the increasing Muslim patient population, more Muslim nurses are needed to help provide religiously and culturally sensitive care to them. More research is needed to understand the mental health and well-being of Muslim nursing students to help with their retention in college, professional development, and longevity in the profession.

To meet this need, the present study attempted to fill in the gap of the research on Muslim nursing students' mental health. Specifically, this study investigates the factors that impact anxiety and depression among Muslim nursing students in Thailand. With a better understanding of their mental health issues, recommendations could be provided to help improve their mental health and well-being.

## 2. Conceptual Framework

**Figure 1:**
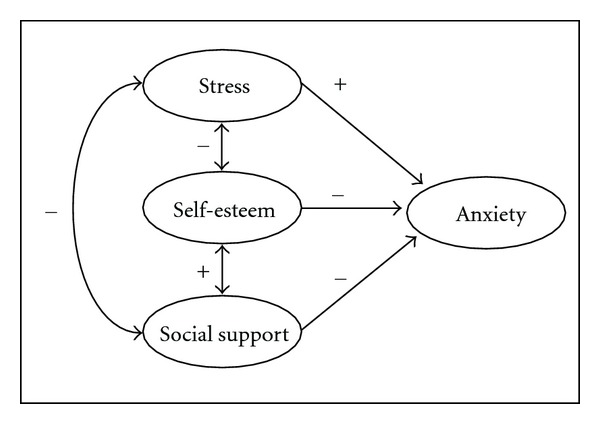
Conceptual framework for anxiety.

Based on previous research, there are two conceptual frameworks for this study. Among Muslim nursing students, stress is hypothesized to be positively associated with anxiety, while self-esteem and social support are hypothesized to be negatively associated with anxiety (see Figure 1). 

**Figure 2:**
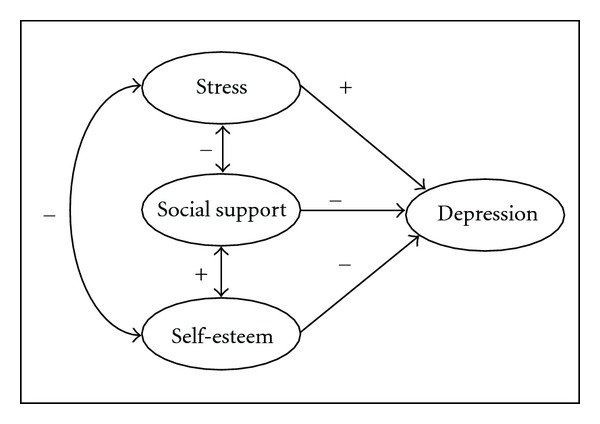
Conceptual framework for depression.

Also among Muslim nursing students, stress is hypothesized to be positively associated with depression, while social support and self-esteem are hypothesized to be negatively associated with depression (see Figure 2).

## 3. Methods

### 3.1. Design and Participants

This cross-sectional research was conducted with a half sampling method of Muslim undergraduate students who were studying at a public nursing college in Thailand. From the 220 Muslim nursing students, half was sampled for this study, representing 14% of the total nursing students at this college. The majority of the nursing students are non-Muslim. The purposes of this research were to investigate the prevalence of anxiety and depression as well as the impact of perceived stress, social support, and self-esteem on anxiety and depression among Muslim nursing students.

The participants for this study consisted of 110 Muslim nursing students. There were 92 females (84%) and 18 males (16%). Participants' age ranged between 20 and 31 years (*M* = 22.8, SD = 2.8). Seventy-nine participants (72%) were third-year students and 31 (28%) were second-year students. All 110 students self-identified as Muslim, with 90 students (83%) describing their Islamic beliefs as very influential in their daily lives and 16 students (15%) describing their Islamic beliefs as moderately influential in their daily lives.

### 3.2. Procedures

The ethics committee approved the study at the nursing college where data collection took place. The survey and informed consent were distributed to half of the Muslim student population on campus and they were invited to voluntarily participate in the study. An incentive of 50 Baht (local currency equivalent to the cost of 2 moderate meals) was given to each participant who completed the anonymous survey.

### 3.3. Instruments

#### 3.3.1. Anxiety

The State Anxiety Scale from the State-Trait Anxiety Inventory was used to measure the participants' level of anxiety [52]. The State Anxiety Scale includes 20 items that assess the current anxiety symptoms using a 4-point Likert scale (0 = not at all, 3 = very much so). A higher score indicates a higher level of anxiety. The State Anxiety Scale has previously been translated into Thai for use in several studies with Thai participants [53]. The internal consistency (Cronbach's alpha) of the State Anxiety Scale for the current sample was  .91.

#### 3.3.2. Depression

The Center for Epidemiology Studies-Depression Scale (CES-D) was used to measure the participants' depressive symptoms [54]. The CES-D consists of 20 items that assess for frequency of the depressive symptoms using a 4-point Likert scale (0 = rarely or none of the time, 3 = most or all of the time). A higher score indicates a higher level of depression. The CES-D has been translated into Thai for use in several studies with Thai participants [39, 55]. The Cronbach's alpha of the CES-D for this study's sample was  .87.

#### 3.3.3. Self-Esteem

The Rosenberg Self-Esteem Scale (RSE) was used to measure participants' self-esteem [56]. The RSE has 10 items including both positive and negative statements about the self, using a 4-point Likert scale (1 = strongly disagree, 4 = strongly agree). A higher score indicates higher self-esteem. The RSE has been translated into Thai and has been used in several previous studies [51, 57, 58]. The coefficient alpha of the RSE for the current sample was  .82.

#### 3.3.4. Perceived Stress

The Perceived Stress Scale (PSS) was used to measure participants' level of perceived stress in the past month [59]. The PSS features 10 items written in both positive and negative form using a 5-point Likert scale (0 = never, 4 = very often). A higher score indicates a higher level of perceived stress. The PSS has been used in several previous studies with college and nursing students [60, 61]. The coefficient alpha of the Perceived Stress Scale for the current sample was  .77.

#### 3.3.5. Social Support

The Multidimensional Scale of Perceived Social Support (MSPSS) was used to measure participants' level of perceived social support from family, friend, and significant other [62]. The MSPSS includes 12 items using 7-point Likert scale (1 = strongly disagree, 7 = strongly agree). A higher score indicates a higher level of perceived social support. The MSPSS has been translated into Thai and has been used in several previous studies [46, 63]. The coefficient alpha of the MSPSS Scale for this study's sample was  .91.

## 4. Results

### 4.1. Descriptive Statistics

Table 1 presents the means, standard deviations, internal consistency estimates, and correlations for the instruments in this study. Several analyses were conducted to examine the differences in the demographic variables and the measured variables. No significant differences in the gender, academic level, and level of religious influence were found.

### 4.2. Prevalence of Anxiety and Depression

According to the score classification for the State Anxiety Scale [52, 64], 26% of the participants in this study were considered anxious. In addition, according to the score classification by Radloff [54], 47% of the participants were found to have at least a mild level of depression.

### 4.3. Predictors of Anxiety

Simultaneous multiple regression analysis was used to assess the impact of self-esteem, stress, and social support on anxiety (see Table 2). Stress (*β* = .42) was positively correlated with anxiety, and self-esteem (*β* = −.42) was negatively correlated with anxiety. Social support was not a significant predictor for anxiety. This model elucidated 46% of the variance in anxiety. Part correlation was used to determine the unique relationship between each predictor variable and anxiety. Stress accounted for 13% of the variance in anxiety; self-esteem accounted for 13% of the variance in anxiety; the collinearity of stress and self-esteem accounted for 22% of the variance.

### 4.4. Predictors of Depression

Simultaneous multiple regression analysis was also used to assess the impact of self-esteem, stress, and social support on depression (see Table 3). Self-esteem (*β* = −.41) and social support (*β* = −.17) were negatively correlated with depression, and stress (*β* = .37) was positively correlated with depression. This model elucidated 57% of the variance in depression. Part correlation was used to determine the unique relationship between each predictor variable and depression. Self-esteem accounted for 12% of the variance in depression, while stress accounted for 10% and social support accounted for 2%; the collinearity of self-esteem, stress, and social support accounted for 36% of the variance.

## 5. Discussion

The results of this study indicated a moderate prevalence of anxiety (26%) and a high prevalence of depression (47%) among Muslim nursing students. These results are consistent with findings from previous research with nursing students and other health professional students. A moderate prevalence of anxiety was found among medical students in Lithuania [41]. A high prevalence of depression was found among nursing students in Thailand [39] and Iran [38]. This present study, however, was the first to investigate the predictors of anxiety and depression among Muslim nursing students. Stress was found to be positively associated with anxiety, while self-esteem was negatively associated with anxiety. Self-esteem and social support were found to be negatively associated with depression, while stress was positively associated with depression.

### 5.1. Implications

**Figure 3:**
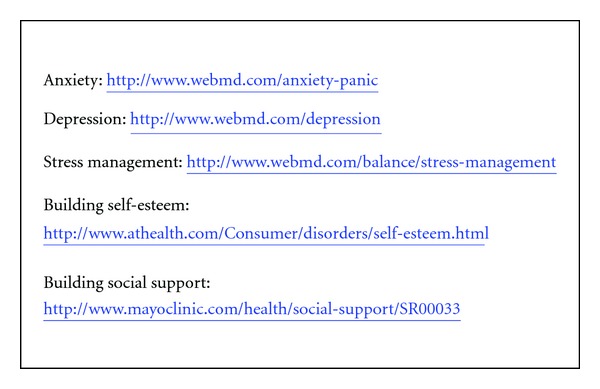
Internet resources for nurse educators and nursing students.

Several implications can be drawn from the results of this study. First, the mental health and well-being of nursing students are very important for nurse educators to address, as anxiety and depression are two of the most common mental health problems that nursing students face. Similar to other nursing students, Muslim nursing students also face a high prevalence of depression and a moderate prevalence of anxiety that must be addressed. In agreement with Kernan and Wheat's recommendations [65] for nurse educators, the first step to helping Muslim nursing students would be to identify emotional issues early and establish working partnership with campus mental health professionals to facilitate referrals for timely intervention. Early diagnosis and treatment are key to helping with Muslim nursing students' retention and graduation. When campus mental health professionals are not available, nurse educators and Muslim nursing students could utilize reliable Internet resources to help identify the signs and symptoms of anxiety and depression as well as pursue treatment recommendations (see Figure 3).

Second, self-esteem and social support are important variables that have been shown to be negatively associated with anxiety and depression among Muslim nursing students in this study. Given these findings, by improving the self-esteem and increasing social support for Muslim nursing students, the resulting decrease in anxiety and depression could be expected. Specific ways to improve Muslim nursing students' self-esteem include becoming aware of needs and wants, starting a self-care habit such as regular exercise, healthy eating habit, and modifying negative thoughts into positive ones. Ways to increase social support for Muslim nursing students include identifying individuals by whom one feels supported and finding ways to increase contact with them; identifying activities that one enjoys doing with others and ways to encourage others to join in these activities; spending more time talking with trusted individuals and not isolating oneself. Previous studies have also recommended a variety of ways to improve self-esteem and social support for nursing students [39, 66]. For additional recommendations, see Figure 3 for internet resources on building self-esteem and social support.

Lastly, stress is the second most important variable after self-esteem in contributing to anxiety and depression among Muslim nursing students. Many forms of stress are normal and functional in assisting students to accomplish tasks, especially stress that students feel when deadlines and examinations are approaching. But if the stress level is too high, it could turn into anxiety or depression. There are many ways for Muslim nursing students to manage stress, including regularly using relaxation techniques such as a deep breathing exercise, maintaining a positive attitude, learning to set appropriate boundaries, and getting enough sleep. One previous study has also recommended intervention to help nursing students manage their stress [67]. For additional recommendations, see Figure 3 for internet resource on stress management.

### 5.2. Limitations and Future Research

Limitations for this study include a sample drawn from only one country and a lack of qualitative data on the experiences of Muslim nursing students. Additional research is needed to further understand the mental health issues among Muslim nursing students from other countries. A qualitative study of the experiences of Muslim nursing students would also provide deeper insight into their mental health, self-esteem, social support, stress, and related issues. Future research should also focus on the impact of stress management, social support enrichment, and self-esteem enhancement programs on the mental health and well-being of Muslim nursing students. Lastly, comparative studies involving nursing students from different faith and cultural backgrounds would further contribute to the field.

## 6. Conclusion

With the growing Muslim population worldwide, it is crucial to recruit and train more Muslim nursing students who recognize and respect their distinct cultural and religious practices to care for Muslim patients. With increasing mental health problems found on college campuses, including in nursing programs, it is imperative that nurse educators and administrators provide more support and resources to help Muslim nursing students do well academically as well as psychologically in order to prepare them for the challenges of the nursing profession. A more psychologically healthy Muslim nursing student will be more likely to graduate in a timely manner, to have a more enjoyable work experience, and to persist longer in the nursing profession.

## Figures and Tables

**Table 1:** Descriptive statistics and correlations between instruments (*N* = 110).

	*M*	SD	*α*	1	2	3	4	5
(1) Anxiety	40.13	8.45	.91	—	.63^∗∗∗^	.59^∗∗∗^	−.27^ ∗∗ ^	−.59^∗∗∗^
(2) Depression	16.29	8.83	.87		—	.62^∗∗∗^	−.48^∗∗∗^	−.65^∗∗∗^
(3) Stress	15.97	4.78	.77			—	−.36^∗∗∗^	−.46^∗∗∗^
(4) Social support	67.22	10.96	.91				—	.44^∗∗∗^
(5) Self-esteem	30.78	4.19	.82					—

**p < .01, ***p < .001.

**Table 2:** Multiple regression analysis for variables predicting anxiety.

Variable	*B*	SE *B*	*β*	*t*	*r* part	*r* ^2^ part
Self-esteem	−0.86	0.17	−.42	−5.07^∗∗∗ ^	−.36	.13
Stress	0.74	0.14	.42	5.19^∗∗∗^	.36	.13
Total unique						.26
Common						.22

Total						.48

Note: adjusted *R* ^2^ = .46, ***p < .001.

**Table 3:** Multiple regression analysis for variables predicting depression.

Variable	*B*	SE *B*	*β*	*t*	*r* part	*r* ^2^ part
Self-esteem	−0.86	0.16	−.41	−5.45^∗∗∗^	−.34	.12
Stress	0.68	0.13	.37	5.04^∗∗∗^	.32	.10
Social support	−0.14	0.06	−.17	−2.39^∗^	−.15	.02
Total unique						.22
Common						.36

Total						.58

Note: adjusted *R* ^2^ = .57, *p < .05, ***p < .001.
